# Unusual Presentation of a Small-Cell Variant of Anaplastic Large-Cell Lymphoma Case: When a Septic Picture Is Not Sepsis

**DOI:** 10.1155/2017/7292148

**Published:** 2017-09-24

**Authors:** Zhou Yu, Yifan Pang, Linda Wang, Daniel E. Ezekwudo, Foluso Ogunleye, Susanna S. Gaikazian, Mark Micale, James Huang, Ann Marie Blenc, Ishmael Jaiyesimi

**Affiliations:** ^1^Department of Hematology and Oncology, William Beaumont Hospital, Royal Oak, MI, USA; ^2^Oakland University William Beaumont School of Medicine, Rochester, MI, USA; ^3^Department of Internal Medicine, William Beaumont Hospital, Royal Oak, MI, USA; ^4^Department of Pathology and Laboratory Medicine, William Beaumont Hospital, Royal Oak, MI, USA

## Abstract

We report a case of a small-cell variant of anaplastic large-cell lymphoma, with an unusual clinical presentation mimicking sepsis and a fulminant clinic course, in a 48-year-old Caucasian female. In this report, we discuss the diagnostic challenge, histopathologic features, and unique cytogenetic features of this case, in order to raise awareness of this rare presentation and emphasize the importance of meticulous peripheral smear examination and early bone marrow evaluation.

## 1. Introduction

Anaplastic large-cell lymphoma (ALCL) is a rare aggressive T-cell lymphoma, comprising 3% of adult-onset non-Hodgkin lymphomas. According to the 2016 WHO classification, ALCL consists of four major groups: primary systemic anaplastic lymphoma kinase-positive (ALK+) ALCL, primary systemic anaplastic lymphoma kinase-negative (ALK−) ALCL, primary cutaneous ALCL, and breast implant-associated ALCL [1]. ALK+ ALCLs exhibit a wide histologic spectrum, including classic type, small-cell, lymphohistiocytic, and sarcomatoid variants [2]. The small-cell variant of ALK+ ALCL comprises 5–10% of cases. Overall, the prognosis of ALK+ ALCL is remarkably better than of ALK– ALCL. However, even though all small-cell variant cases have been shown to be ALK+, the prognosis is very poor for this subgroup [3, 4]. Here, we present a case of ALK+ ALCL with small-cell variant morphology.

## 2. Case Presentation

A 48-year-old previously healthy Caucasian female presented with progressive shortness of breath on exertion, right upper quadrant abdominal pain, and left lower extremity pain and swelling. On presentation, the patient was afebrile; however, she was borderline hypotensive, tachycardic, and tachypneic. Laboratory results showed marked leukocytosis (37.3 × 10^9^/L) with predominant neutrophilia and mild thrombocytopenia, as well as acute kidney injury, elevated transaminases with hyperbilirubinemia, and metabolic acidosis.  Table 1 summarizes the relevant laboratory findings. Initial differential diagnosis included sepsis, cholecystitis, or pulmonary embolism. Empiric broad-spectrum antibiotics were initiated immediately. Imaging studies did not show any venous thromboembolism or cholecystitis but revealed hepatomegaly, small lung nodules, and mild axillary lymphadenopathy.

**Figure 1:**
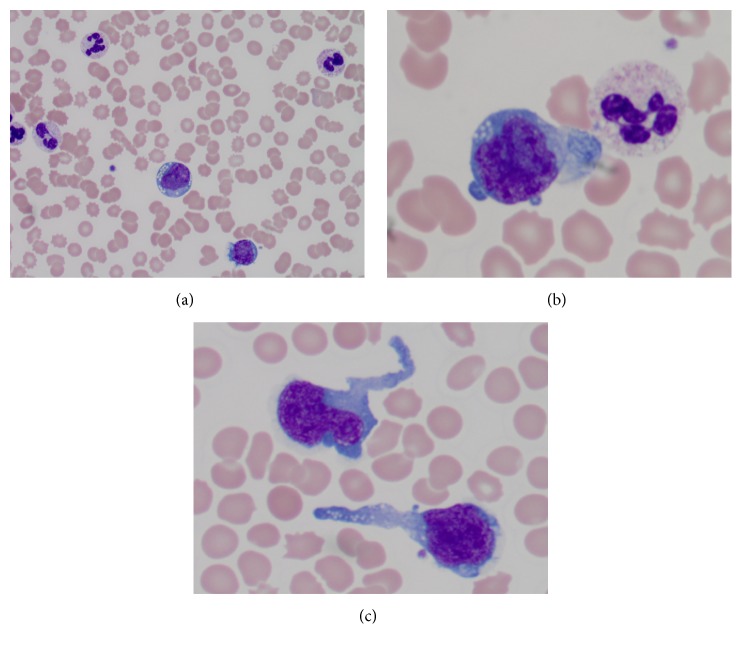
Peripheral blood examination. (a) The patient's peripheral blood smear shows leukocytosis with neutrophilia, left shift, and toxic granulation (Wright's stain, 40x). (b) Scattered medium size atypical cells are present. These cells feature prominent nuclear irregularities and vacuoles (Wright's stain, 100x oil). (c) Atypical cells have pseudopodial cytoplasmic projections (Wright's stain, 50x oil).

**Figure 2:**
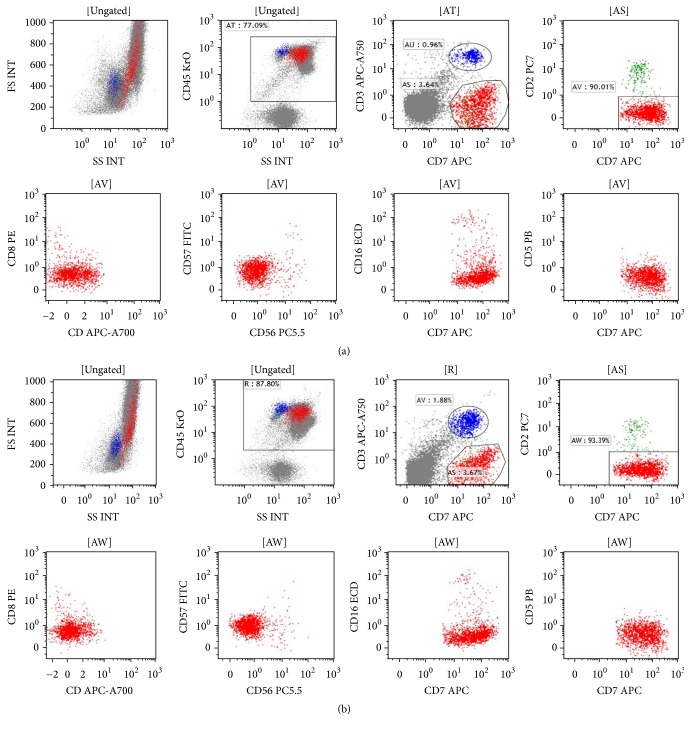
Peripheral blood and bone marrow aspiration analyzed by flow cytometry. Flow cytometry analysis of peripheral blood (a) and bone marrow (b) revealed a similar small population of atypical lymphocytes (red) which are positive for CD45 and CD7 and negative for CD2, CD3, CD4, CD5, CD8, CD16, CD56, and CD57. Normal T-cells are in blue and normal natural killer cells are in green.

**Figure 3:**
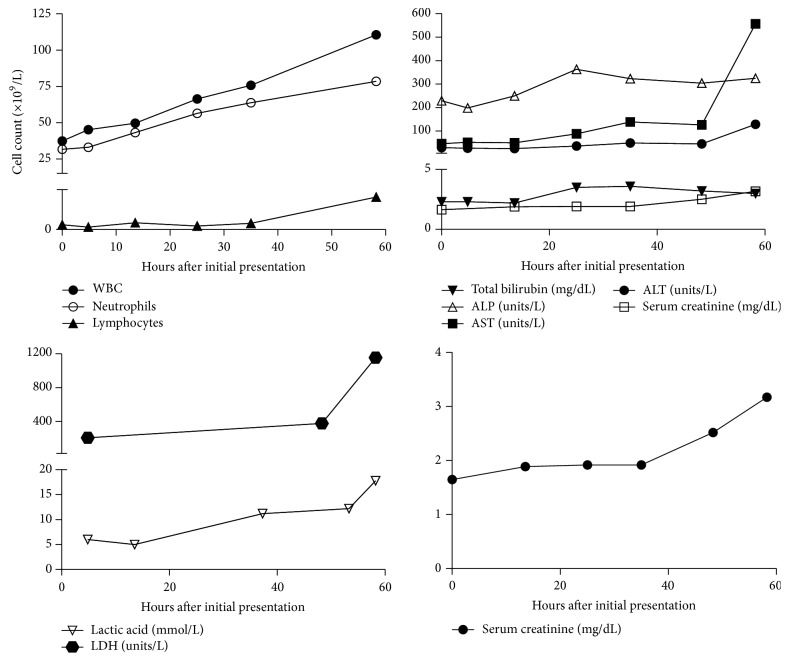
Laboratory parameters along the patient's clinical course. White blood cells (WBC), absolute neutrophil counts, lymphocyte counts, renal and liver function tests, and lactic acid and lactate dehydrogenase (LDH) levels are shown.

Extensive infectious workup studies including urine culture, blood culture, respiratory virus panel, hepatitis, and HIV were all negative. The peripheral blood smear showed severe neutrophilia with toxic granulation and a small number of atypical lymphocytes (Figure 1). These atypical cells have an irregular nuclear contour and prominent nucleoli and vacuoles. Hematolymphoid malignancies were added to the differential diagnosis list. Flow cytometric analysis of peripheral blood (Figure 2(a)) revealed a small population of abnormal T-cells, which was positive for CD45 and CD7, but negative for CD2, CD3, CD4, CD5, CD8, CD16, CD56, and CD57. In addition, aberrant CD13 expression was also detected (data not shown). These cells featured increased forward scatter and side scatter, when compared with immunophenotypically normal lymphocytes. While pathological evaluation of the bone marrow was in process, the patient's leukocytosis rapidly worsened and peaked at 110.6 billion/L. She developed multiorgan failure requiring ventilator support, continuous renal replacement therapy, and multiple vasopressors. Her laboratory parameters along her clinical course are shown in Figure 3. Despite maximum supportive therapy, her condition continued to decline. After extensive discussion, her family decided to pursue comfort care and she expired approximately 80 hours after initial presentation.

**Figure 4:**
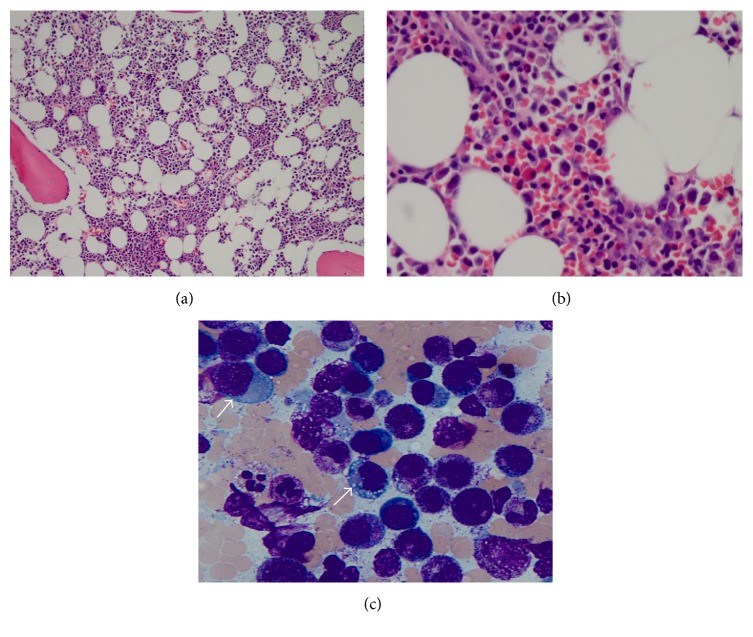
Morphological features of bone marrow core biopsy and aspiration. The H&E* section of* bone marrow core biopsy demonstrated hypercellularity for the patient's age with myeloid hyperplasia ((a) 100x magnification; (b) 400x magnification). Scattered lymphoma cells (pointed by the white arrows) were easily seen on aspirate smears ((c) Wright's stain, 1000x magnification) but not easily discernible on H&E sections.

**Figure 5:**
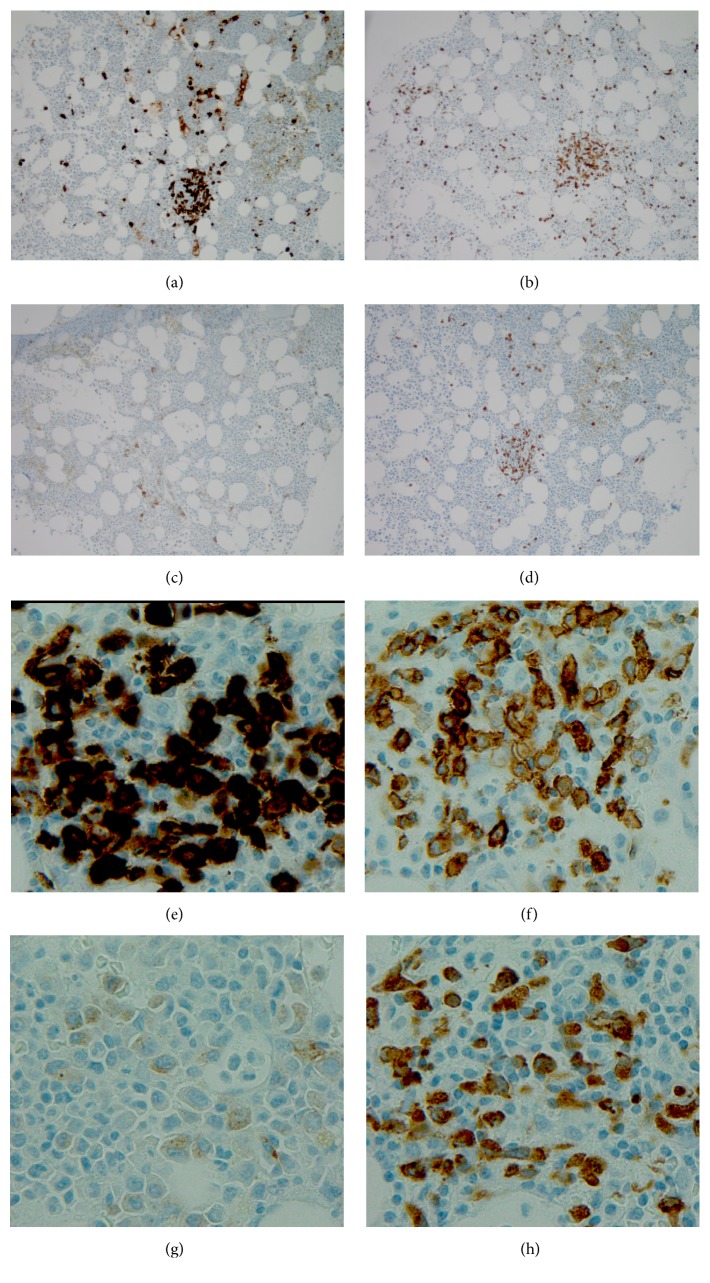
Immunohistochemistry staining of bone marrow core biopsy. Small to intermediate sized neoplastic cells form loose clusters and comprise 5–10% of marrow cellularity. These cells stain positive for CD30 (a, e), CD7 (b, f), EMA (equivocal) (c, g), and ALK (d, h). (a–d) 100x magnification; (e–h) 1000x magnification.

**Figure 6:**
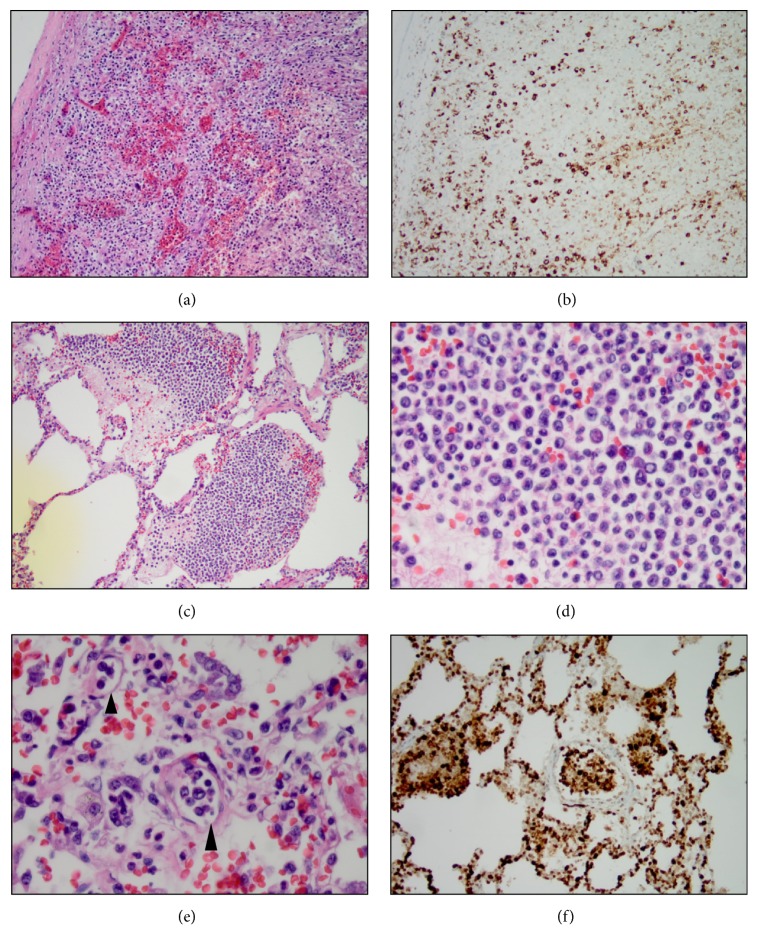
Representative autopsy sections from spleen and lung. Lymphoma cells were not overtly discernible on H&E section ((a) 100x magnification) but clearly identified by immunostains ((b) CD30 staining) in the spleen. In the lung parenchyma, lymphoid infiltrates were prominent with predominantly small to medium sized cells (H&E staining; (c) 100x magnification; (d) 400x magnification). Intravascular lymphoma involvement was evident morphologically ((e) H&E staining, 400x magnification) and confirmed by immunostains ((f) CD30, 200x magnification). Black arrowheads in (e) refer to intravascular lymphoma involvement.

**Figure 7:**
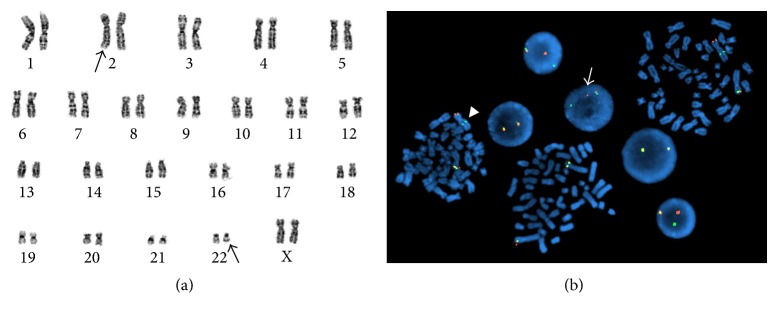
Cytogenetic analysis of bone marrow. (a) The G-banded metaphase cell demonstrates abnormalities involving chromosomes 2 and 22 (black arrows), interpreted to represent an initial chromosome 2 pericentric inversion that disrupts the ALK gene at band 2p23 and translocates the centromeric portion of the gene to band 2q22. This is followed by a translocation between the inverted chromosome 2 (with a breakpoint distal to the ALK gene) and chromosome 22q. The karyotype is designated as 46,XX,inv(2)(p23q22)t[inv(2);22)(p24;q12)]. (b) Fluorescence in situ hybridization (FISH) utilizing an ALK gene break-apart probe demonstrates disruption of the gene (white arrow) and relocation of the centromeric portion of the gene to the long arm of chromosome 2 (white arrow head) as a result of the chromosome 2 pericentric inversion.

The flow cytometric analysis of the bone marrow aspirate (Figure 2(b)) showed a small T-cell population (3.7% of marrow nucleated cells) with identical immunophenotypic features to those in peripheral blood. Bone marrow core biopsy revealed hypercellularity with loose clusters of small to medium sized neoplastic cells, accounting for 5–10% of the marrow cellularity (Figures 4 and 5). The immunohistochemistry studies of the core biopsy revealed that lymphoma cells expressed CD30 (strong), CD7 (strong), and ALK (cytoplasmic) (Figure 5). The expression of epithelial membrane antigen (EMA) appeared to be equivocal. Autopsy showed diffuse microscopic lymphoma infiltration of the spleen (Figures 6(a) and 6(b)), lung (Figures 6(c) and 6(d)), and liver. Intravascular lymphoma involvement was present in both brain and lung (Figures 6(e) and 6(f)). Bone marrow cytogenetic evaluation identified an unusual chromosome 2 inversion [inv(2)(p23q22)] resulting in ALK gene rearrangement, as well as a translocation involving the inv(2) chromosome with chromosome 22q (Figure 7(a)); however, fluorescence in situ hybridization (FISH) studies demonstrated that the ALK gene was not involved in the translocation with chromosome 22 (Figure 7(b)). These findings are consistent with small-cell variant of ALCL.

## 3. Discussion

Prognosis in ALK+ ALCL is good overall, except for the small-cell variant [3, 5]. Correct clinical diagnosis of the small-cell variant of ALCL is often challenging as the scarce “hallmark cells” are scattered among inflammatory cells and may be difficult to recognize.  Table 2 lists characteristics of small-cell and classic variants of ALCL. The extraordinary leukocytosis and “left shift” often prompt an extensive microbiology and serology workup in search for a cause of presumed infection or sepsis. Mosunjac et al. reviewed 23 autopsy cases of ALCL and reported a distinct subset of 5 fatal and premortem unrecognized ALCL cases [7]. Similar to our case, these cases were characterized by unusual presentations of fever of unknown origin (FUO), sepsis, hepatosplenomegaly, lactic acidosis, rapid clinical deterioration, and absence of significant peripheral lymphadenopathy. These characteristics are different than the classic presentation of ALCL, which includes night sweats, weight loss, and peripheral lymphadenopathy. Recognition of a combination of clinic pictures with markers of sepsis, lactic acidosis, hepatosplenomegaly, and a negative infectious workup should trigger suspicion to rule out ALCL. A meticulous examination of peripheral blood smears, comprehensive immunophenotypic studies, and early bone marrow and lymph node biopsy are needed to facilitate diagnosis. In our case, peripheral smear evaluation revealed atypical lymphocytes which raised suspicion for lymphoma and prompted early bone marrow evaluation. However, the patient's rapid deterioration precluded further lymph node biopsy.

About 60% of ALCL cases are associated with chromosomal translocations involving ALK on chromosome 2p23 [8], which encodes a transmembrane receptor tyrosine kinase that belongs to the insulin receptor superfamily. ALK signaling can be activated by the oncogenic fusion of the ALK gene with a variety of partner genes through translocation events. The constitutive activation of chimeric ALK fusion proteins leads to complex signaling transduction pathways including the JAK/STAT and PI3K/AKT pathways, which control cell proliferation, survival, and cell cycling [8].

Among ALK+ ALCLs, about 80% of cases contain the t(2;5) (p23;q35) translocation, resulting in the formation of an NPM-ALK fusion protein, a transmembrane tyrosine kinase receptor [9]. Variant translocations involving ALK and other partner genes on chromosomes 1, 2, 3, 17, 19, 22, and X also occur [9]. Rare ALK gene rearrangement variants involving inv(2)(p23;q35), resulting in ATIC-ALK fusion, have also been reported previously [10]. The cytogenetic abnormality in our patient with inv(2)(p23q22) has never been reported previously. Because FISH studies confirmed that ALK did not translocate to chromosome 22, the translocation in this patient appears to be dissimilar to the t(2;22) reported in the literature for ALCL. Therefore, ALK rearrangement in this case would appear to generate a novel ALK fusion gene.

While leukemic peripheral blood involvement is rare in ALCL, an association has been reported with small-cell variants [5, 6, 11], which may be a potential explanation for the poor prognosis and aggressive nature of small-cell variant ALCL. Extreme leukocytosis involving neutrophils and atypical lymphocytes is usually the feature noted in the peripheral blood. The neutrophils are often left shifted with toxic granulation features, whereas the lymphocytes are a mixture of small to medium size atypical cells, with an irregular nuclear contour and cytoplasmic azurophilic granules. Large basophilic vacuolated cells can occasionally be seen as well [5].

Another unique feature of our case is the rare intravascular pattern of lymphoma involvement in this presentation of ALK+ ALCL. The vast majority of reported cases are in B-cell lineage lymphomas, and intravascular lymphoma involvement is rarely seen in T-cell lymphoma. Among the small number of cases reported in T-cell lymphoma, the majority of these are peripheral T-cell lymphoma [12, 13]. A review of the literature demonstrated only 11 documented cases of CD30-positive T-cell lymphomas, suggestive of ALCL as the lymphoma type [7, 13–18]. Intravascular involvement was seen in all 5 autopsy cases reported by Mosunjac et al., all of which were ALK-negative, except one [7]. Other than this case described by Mosunjac et al. and our case, only one other ALK+ ALCL case, which mimicked inflammatory breast carcinoma, was reported to have intravascular involvement in the reported English literature [18]. It has been postulated that systemic symptoms and the aggressive course of the disease might be related to release of cytokines. Direct interaction of vascular endothelial cells and lymphoma cells may trigger the proinflammatory cytokine cascade and distort hemostatic balance. Intravascular involvement may be an indicator for poor prognosis as seen in our case and is also suggested by previously reported cases [7].

Despite the generally favorable prognosis of ALK + ALCL, cases with peripheral leukemic involvement often respond poorly to standard first-line anthracycline-based multiagent chemotherapy. Brentuximab vedotin (Adcetris), a CD30 antibody-drug conjugate, has been approved for relapsed or treatment refractory ALCL cases. A Phase III clinical trial is underway to evaluate brentuximab vedotin combined with chemotherapy in the first-line treatment of ALCL (clinical trial number NCT01777152). Crizotinib and ceritinib, ALK tyrosine kinase inhibitors, have shown encouraging activity in small case series [19, 20]. These agents are currently being explored in ongoing clinical trials. Hopefully, these novel therapies may improve the prognosis for ALK+ ALCL variants.

## 4. Conclusion

We report a case of ALK+ ALCL small-cell variant with a presentation mimicking sepsis. Small-cell variant, leukemic peripheral involvement, and intravascular involvement are associated with unfavorable prognosis.

## Figures and Tables

**Table 1:** Laboratory parameters on admission.

Laboratory tests	On admission	Reference range
Total WBC (×10^9^/L)	37.3	4–10
Hemoglobin (g/dL)	12.7	12–15
Platelet (×10^9^/L)	127	150–400
Neutrophil (×10^9^/L)	31.7	1.6–7.2
Lymphocyte (×10^9^/L)	1.8	1.1–4.0
Serum creatinine (mg/dL)	1.65	0.6–1.4
Serum BUN (mg/dL)	31	8–22
Alkaline phosphatase (U/L)	229	30–110
AST (U/L)	46	10–37
ALT (U/L)	29	8–37
Bilirubin (mg/dL)	2.3	0.3–1.2
Direct bilirubin (mg/dL)	1.2	0–0.3
Lactic acid (mmol/L)	6	0.5–2.2
LDH (U/L)	207	100–238

WBC: white blood cells; BUN: blood urea nitrogen; AST: aspartate aminotransferase; ALT: alanine aminotransferase; LDH: lactate dehydrogenase.

**Table 2:** Comparison of classic and small-cell variant ALCL.

	Classic ALCL variant	Small-cell ALCL variant
Frequency	70% of ALCL	5%–10% of ALCL

ALK rearrangement	ALK-positive and ALK-negative	ALK-positive

Morphology	Characterized with numerous large round or pleomorphic cells with horseshoe-shaped nuclei with multiple (or single) prominent nucleoli; these cells are also called “hallmark” cells	The majorities of cells are small to medium in size and have clear cytoplasm and irregular nuclei; a minor population of “hallmark” cells can present singly or in cluster [5]

Peripheral leukemia involvement	Extremely rare	Very common [5, 6]

Bone marrow involvement	Relatively infrequent (10%–30% of cases)	Common; usually subtle bone marrow involvement with a small cluster of small lymphocytes and only rare scattered large tumor cells [5, 6]

Systemic involvement	Relatively less infrequent	Frequently associated with widespread disseminated disease [6]

Prognosis	2-year survival of 73%	2-year survival of 50% and 3-year disease-free survival of 25% [3, 5]
